# CoWIN twitter dataset: A comprehensive collection of public discourse on India's COVID-19 vaccination platform

**DOI:** 10.1016/j.dib.2024.111252

**Published:** 2025-01-02

**Authors:** Shubham Mittal, Swarnalakshmi Umamaheswaran

**Affiliations:** a Symbiosis Institute of Business Management, Symbiosis International (Deemed University), Bengaluru 560100, India; b Mckinsey, Gurugram 122018, India

**Keywords:** CoWIN, COVID-19, Social media analytics, Digital health, Sentiment analysis, Health informatics, Twitter data, India

## Abstract

The CoWIN Twitter Dataset offers a wide-ranging collection of public opinions on India's COVID-19 vaccination platform CoWIN. The raw dataset has 635,000 tweets that mention “cowin,” collected over the period of January to December 2021. The dataset was extracted by employing the Twitter Academic API. It addition to the raw data, it also included a cleaned and processed set of 419,409 English tweets, and a labeled subset with sentiment analysis. The raw data file has tweet details like ID, text, timestamp, user ID, and language. The processed dataset is devoid of URLs and hashtags and other noise, and also adds month and category groupings. Finally,the labelled dataset gives sentiment classifications of positive or negative the relevant tweets. This dataset enables researchers to analyse themes and sentiments related to India's vaccination administration. It can help policymakers gain insights around issues related to large-scale health initiatives and digital health systems. The mix of languages in the data also makes it useful for language processing research.

Specifications Table

**Table:** 

Subject	Social sciences.
Specific subject area	Application of NLP methods for extracting themes and sentiment towards the cowin app).
Type of data	Raw text Additional files created after data cleaning, filtering and processing
Data collection	Data was collected through APIs from the social media platform twitter (now X). Tweets were extracted using the official API meant for academic research using the specific keyword ‘cowin’. The package TwitteR was used to interface with the API.
Data source location	Country: India.
Data accessibility	Repository name: Mendeley Data identification number: (10.17632/k5yr89ms8s.2) Direct URL to data: https://data.mendeley.com/datasets/k5yr89ms8s/1
Related research article	None

## 1 Value of the Data

• This dataset is helpful in understanding themes, stakeholders and the sentiment around the usage of CoWin app, how it evolved through India's vaccination programme. Insights from the data can help policymakers and public health officers plan large scale health initiatives.
• Researchers across social sciences can use this data to analyze public sentiment towards digital health platforms and also track the evolution of opinion over time. It can provide insights on technical and functional issues around vaccination distribution and administration.
• The dataset can be used as a benchmark dataset by computational linguistic researchers given its multilingual nature and the availability of mixed language use, sarcasm and other linguistically valuable features. In this context, the dataset is an important contribution to existing linguistic corpora for advancing natural language
• While there exists several datasets on Covid 19, this data offers a unique perspective on the technology augmented administration of Covid vaccines. In this context it also complements existing structured datasets around Covid 19 vaccination
• The dataset and the accompanying experiment demonstrate the sentiment generation for text in low resource settings by employing pre-trained models, highlighting their potential in similar contexts

## 2 Background

India's COVID vaccination program is notable for two key reasons. First, it was undeniably the world's largest immunization campaign with the intent of covering 1.3 billion citizens [1]. Second, it was entirely driven by technology. As of date, 88 % of the population is fully vaccinated, which is significantly higher than the global average of 69 % [5]. The technology aspects of the campaign are of particular interest, since it was beset with several teething problems, both functional and technical. The vaccination drive was managed entirely using IT applications - mainly the Co-Win platform. The platform had modules for registering and scheduling vaccination for beneficiaries, a facilities module which maintained information about vaccination centres and hospitals, a vaccination module which captured the event for every individual, and finally a certificate module which maintained the vaccination certificates [6]. However, the Co-Win platform was beset with glitches, both technical and functional [8], and the design itself co-evolved along with the immunization program. In this sense, a study of Co-Win and the documentation of user perception and issues can provide valuable knowledge for product design in similar health care use contexts.

## 3 Data Description

This dataset, accessible via the Mendeley data repository, comprises three files. Each file serves a specific analytical purpose, providing a comprehensive overview of the public discourse surrounding the CoWIN app as expressed on Twitter. The files are systematically organized to facilitate ease of access and detailed analysis, with each file targeting a different stage of data processing.

1. Raw Data (CoWin_raw_2021.csv):

This file has an extensive collection of 635,000 tweets harvested from January to December 2021 through the Twitter Academic API. It includes all tweets that mentioned the keyword “cowin” regardless of context or relevance, thereby providing a raw snapshot of the discussion. Since CoWin was an open source platform made available for several countries multilingual tweets are a part of this dataset.

The Document contains abundant metadata, including categories like - Tweet_ID, Text, Timestamp, User_ID and Language. This Extensive Metadata compilation enables varied approaches to analysis such as language based or country specific investigations. Table 1 displays the language code in twitter and its interpretation. We observe multiple Indian regional languages along with south Asian and a few European languages.

**Table 1:** Language codes in the dataset.

En	English
Tl	Tagalog
hi	Hindi
Ro	Romanian
In	Indonesian
Et	Estonian
Und	Undetermined
Fr	French
Qme	for tweets with media links
Mr	Marathi
Zxx	tweets with either media or Twitter Card only, without any additional text
Ht	Haitian
Ta	Tamil
Ja	Japanese
Qu	Gujarati

**Fig. 1:**
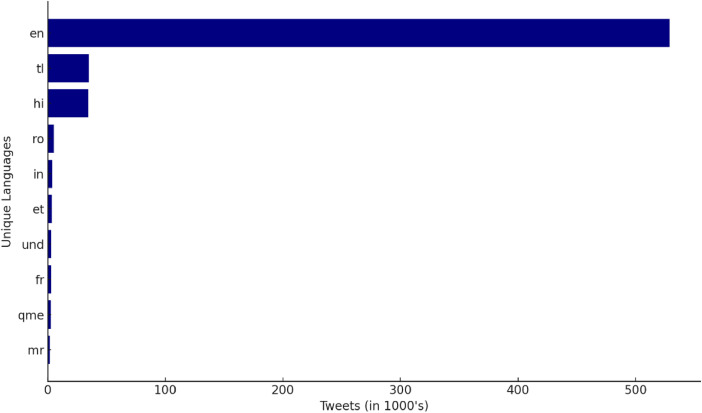
Language distribution of CoWin tweets.

English however is the dominant language as shown in Fig. 1.

2. Processed Corpus (tweets with sentiments.csv):

The second file is derived from raw data after removing all non-English tweets and deduplication. Specifically, it has 470,853 English tweets that were most relevant and best suited for in-depth analysis.

Additionally, we have augmented the original data with two columns: Month, which determines the month from original tweet timestamp and Category, which classifies tweets as informational capturing public health announcement, logistics update regarding vaccines, etc. We also have two columns of redicted sentiment inferred by the pre-trained models in out experiment.

**Fig. 2:**
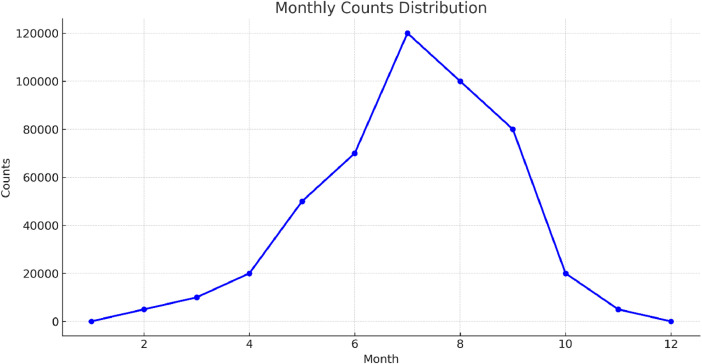
Distribution of tweets by month.

**Fig. 3:**
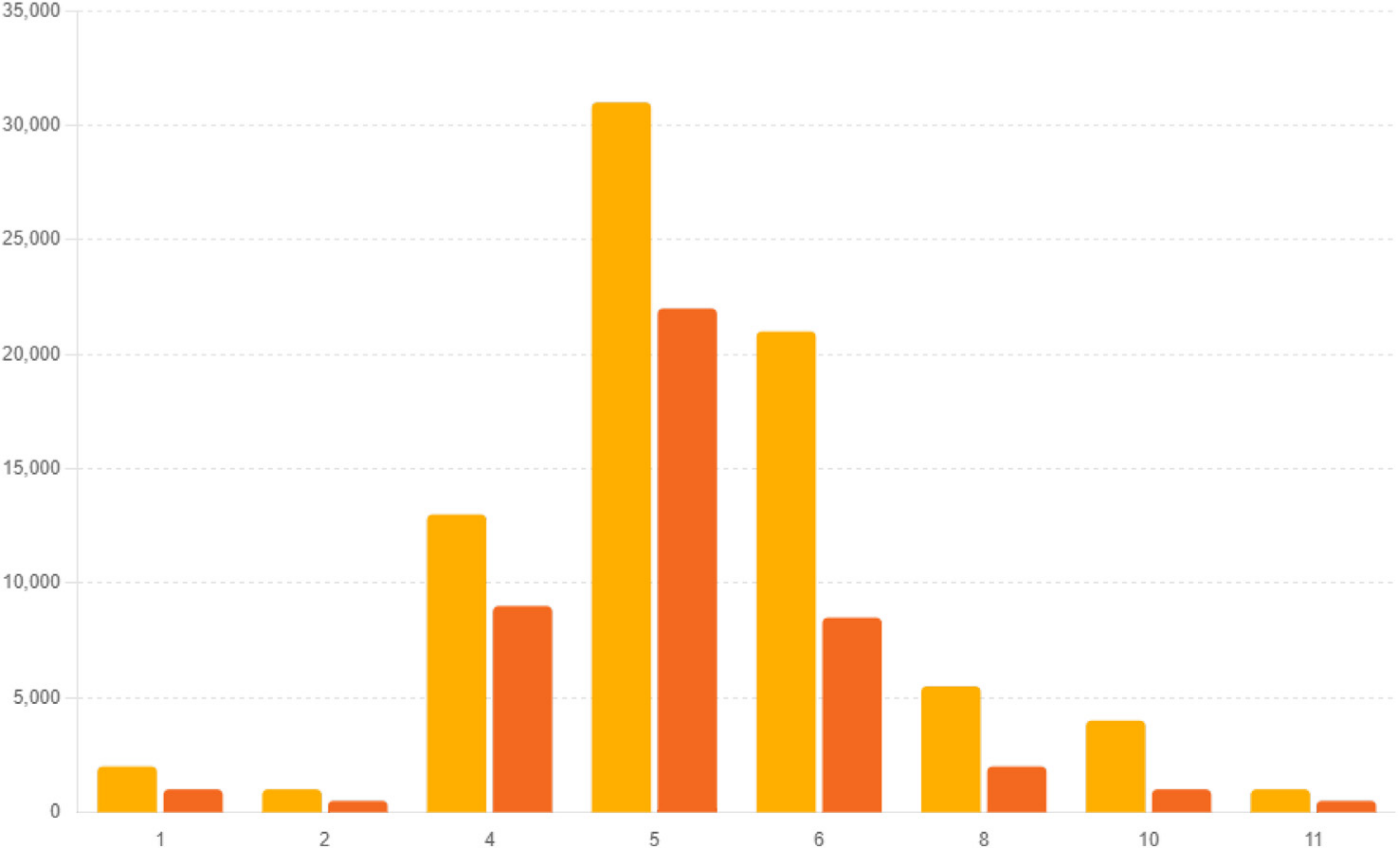
Count of positive and negative sentiments by month.

Fig. 2 exhibits a histogram illustrating the distribution of tweet counts per month. This graphical depiction monitors the changes in discussion activity happening throughout the year, offering insights into peak engagement periods and potential correlated events that occur (Fig. 3).

## 4 Experimental Design, Materials and Methods

### 4.1 Data collection

**Fig. 4:**
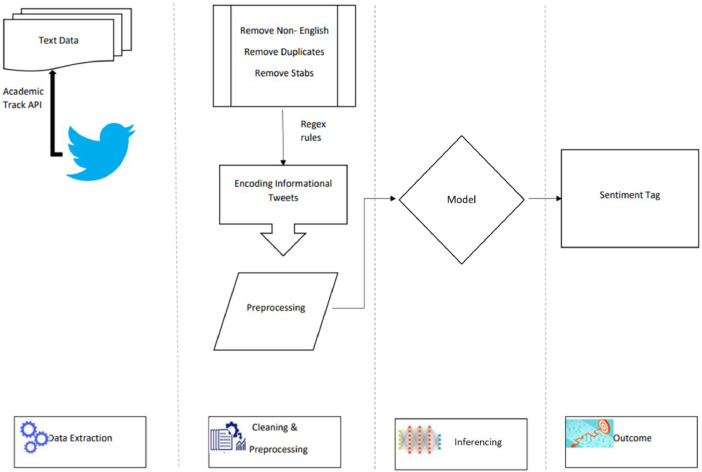
Workflow for the data curation and processing.

We collected the data through the Academic research product introduced by twitter in 2021.It included features such as full access to the archive and higher rate limit of 10 million tweets a month, For our investigation we specifically queried the Twitter API to fetch tweets that mentioned "cowin" in a case-insensitive manner, ensuring comprehensive coverage of the discourse. Tweets were collected in JSON format, which retains all metadata associated with each tweet, providing a rich dataset for analysis. This method enabled the capture of a broad range of tweets, from public opinions and personal experiences to official updates related to the CoWIN platform. The dataset had multilingual tweets with English contributing to 80 % of the tweets. For this investigation we focused only on English tweets for the following reasons. First, English clearly was the dominant language in the dataset. Second, the structure of non-English languages could vary significantly. For instance Indian language tweets are often characterized by code mixing where more than one language is detected in the same statement. Code-mixing is a salient problem and requires custom preprocessing and algorithms differentiated for varied combinations of languages Third, as outlined above validation based on human annotated sample was a key aspect of our methodology. However human annotation in non- English languages was a large scale and resource intensive exercise which was beyond the scope of this study (Fig. 4).

### 4.2 Data preprocessing

Data processing is crucial for tweets since their textual content ranged from formal to informal and may contain symbols such as emoticons, numbers, dates etc. In the case of tweets it is common to find URLs, mentions and hastags as well. Deciding what to remove and what to retain was a key challenge. We opted to delete all user mentions in tweets in order to preserve privacy. While URLs, hashtags and numbers are often recommended to be removed since they do not directly deliver meaning [3,4], In this case however, we reconsidered their role based on a qualitative examination. We recognized the additional context they may provide for transformer based algorithms and retained them in the dataset.

As part of the data processing step we also added new features to the dataset. First is the extraction of the month from the date time format. We also added a new column flagging tweets which where announcements regarding vaccine distribution as informational. These tweets were typically posted by health care organizations announcing vaccine slots and location tagged with the pin code. A few instances of such tweets are below

“Pin Code:[411004] Galaxy Care Multispec PMCP Vaccines: COVISHIELD, Min Age Limit: [45,18], Dose1 Capacity: 0,319,361, Dose2 Capacity: 3,0,1, Dates Available: 02-07-2021,03-07-2021 #VACCINE #VACCINEPUNE #COWIN #COVID19 #PUNE”

“&lt;&lt;&lt;Vaccine Alert&gt;&gt;&gt; Date: 11-08-2021 Pincode: 400071 Center Name: Sushrut Hospital 1 Minimum Age: 18 Vaccine: COVAXIN Dose 1: 40, Dose 2: 86 Fee type: Paid #COWIN #VACCINE #COVID19 #VaccinateMumbai”.

It can be clearly observed that these tweets are factual and they did not focus on any specific problems or features of CoWIN. Consequently they do not directly express sentiment and hence cannot be tagged as positive or negative. However they may shed light on other aspects of the discourse such as vaccine availability and the operational aspects of the distribution system hence we considered them as valuable. We use regular expression with targeted keywords such as ‘vaccine alerts’, ’available capacity’ and other information such as zip code variations, dose, age and availability details to flag tweets as informational.

### 4.3 Sentiment labels

For generating sentiments, we considered pre-trained algorithms. The advantage of using pre-trained models in automated labeling and prediction in resource-constrained environments is well-supported in other studies [7]. In our study context, we evaluated two algorithms. Our choice of algorithms was guided by their relevance to our dataset. We chose the sentiment classifier trained on COVID-19 pandemic data, making it relevant for analyzing CoWIN-related tweets. In contrast, we then chose a RoBERTa-base model for its high performance in general-purpose Twitter data, making it robust for all types of tweets.

Our first classifier, the Lampert and Lampert [2], used emoticons associated with tweets as labels for sentiment and classified tweets as either positive or negative across multiple languages. The training dataset for the model contains 5 million tweets related to the pandemic. Despite its multilingual capabilities, this pre-trained model achieved the highest accuracy in English tweets, with an AUC of 0.77 [2], and hence was well-suited for English language corpora. The second algorithm is the Twitter-RoBERTa-base model, which is optimized to classify tweets as positive, neutral, and negative. The model is a fine-tuned RoBERTa-based model trained on 58 M tweets, which was later extended to 124 M tweets covering the period of 2018–2021. The model is highlighted for its accuracy in classifying informal language – a characteristic feature of tweets. The model reported a recall of 73 % when benchmarked against the TweetEval dataset and integrates as part of the TweetNLP web-based application for social media analysis [9].

To evaluate the accuracy of our labels, we tested the model on a human-annotated sample. To determine the size of a statistically significant sample, we used Cochran's formula, which estimated the minimum sample size to be 384. We further expanded this to a random sample of 500 tweets, which is well above the minimum sample size required for statistical power. Three human annotators, based on the sentiment expressed, labeled each tweet as “negative” or “positive.” In order to evaluate the inter-rater agreement, we calculated Krippendorff's alpha, which at 0.82 indicated a fairly high level of agreement between raters. Wherever there was disagreement between raters, we used the majority voting approach to resolve the final label.

We then compared the final label with the predicted labels generated from RoBERTa-base and Lampert. To evaluate the model's performance, we also generated a confusion matrix to analyze true positives (TP), true negatives (TN), false positives (FP), and false negatives (FN) for each sentiment class. This provided insights into the model's ability to correctly classify positive and negative sentiments. The recall, precision, and F-score for both models are reported below (Table 2, Table 3).

**Table 2:** Performance of models.

Metric	Roberta-Base	Lampert & Lampert
Recall	0.78	0.82
Precision	0.80	0.83
F1-Score	0.77	0.82

**Table 3:** Bootstrapping results for recall and precision metrics.

Metric	Roberta-Base		Lampert & Lampert	
	Mean	Std.dev	Mean	Std.dev
Recall	0.78	0.07	0.83	0.06
Precision	0.80	0.06	0.84	0.06
				

However, given that the annotated data sample is relatively small, it was important to evaluate the robustness of our model's performance. For this, we used bootstrapping to construct confidence intervals around the accuracy scores. This approach involved repeated sampling from the annotated dataset, effectively simulating larger data sizes. Specifically, we selected 500 random samples from the annotated dataset, with each sample consisting of 30 tweets. Through these 500 iterations, we calculated the mean and standard deviation for each recall and precision, which captured the expected variability of these metrics across different samples. The results of the bootstrapping exercise are given below.

From the results we see that the performance of both the models is comparable although Lampert & Lampert slightly outperforming RoBERTa in both precision and recall. Regardless, as compared to models trained from scratch we observe that performance is relatively lower with room for improvement. From a methodological perspective our primary goal was to explore and evaluate the pre trained models in resource constrained environment and hence finetuning was beyond the scope for the experiment. However it would be beneficial for future research to adopt fine tuning strategies and evaluate how model performance improves when trained on CoWIN specific data.

## Limitations and future research

While the study offers a compressive dataset of CoWIN related tweets it also has some limitations. First is exclusive focus on English tweets excluding other Indian and international languages. As a result, sentiments expressed in other languages have not been represented in the dataset. Second are the limitations emerging from the choice of sentiment models., The sentiment labels in the dataset are generated by pre-trained models without fine tuning for CoWin specific contexts. As a result, the sentiment labels may not fully reflect the nuances of the expressions related to coWin. Additionally the study is limited only two models due to its scope. Considering these limitations future research studies can work on finetuning models and expand the availability of human annotated data. Studies can also broaden the experiments to include several models including multilingual transformer based models and compare and evaluate them in terms of performance Further the model's performance on complex expressions, irony sarcasm and multiple sentiments can also be an interesting line of inquiry. Other avenues for reusing this dataset includes, longitudinal analysis of sentiment specifically as a response to key events. policy changes related to vaccination and introduction of new features of CoWIN app itself. Further research can also reuse the dataset to study Vaccination hesitancy, misinformation etc.

## Ethics Statement

Participant data has been fully anonymized, and was curated and distributed as per the Twitter Developer Policy 2021].Individual names and tags were also removed from the tweens in order to maintain privacy. The study only involved publicly available tweets and there was no direct interaction with the individuals who posted the tweets hence public consent was not required.

## CRediT authorship contribution statement

**Shubham Mittal:** Data curation, Writing – original draft. **Swarnalakshmi Umamaheswaran:** Conceptualization, Data curation, Formal analysis, Writing – original draft.

## Data Availability

Mendeley DataCoWin_tweets_2021 (Original data)
